# Prognostic models for intracerebral hemorrhage: systematic review and meta-analysis

**DOI:** 10.1186/s12874-018-0613-8

**Published:** 2018-11-20

**Authors:** Tiago Gregório, Sara Pipa, Pedro Cavaleiro, Gabriel Atanásio, Inês Albuquerque, Paulo Castro Chaves, Luís Azevedo

**Affiliations:** 1 Department of Internal Medicine, Vila Nova de Gaia Hospital Cente, Rua Conceição Fernandes, 4434-502 Vila Nova de Gaia, Portugal; 2 Stroke Unit, Vila Nova de Gaia Hospital Center, Rua Conceição Fernandes, 4434-502 Vila Nova de Gaia, Portugal; 3 Intensive Care Department, Algarve University Hospital Center, Rua Leão Penedo, 8000-386 Faro, Portugal; 4 Department of Internal Medicine, São João Hospital Center, Alameda Prof. Hernani Monteiro, 4200-319 Porto, Portugal; 5 Stroke Unit, São João Hospital Center, Alameda Prof. Hernani Monteiro, 4200-319 Porto, Portugal; 6 Department of Surgery and Physiology, Faculty of Medicine, University of Porto, Alameda Prof. Hernani Monteiro, 4200-319 Porto, Portugal; 7 Center for Health Technology and Services Research & Department of Community Medicine, Information and Health Decision Sciences, Faculty of Medicine, University of Porto, Alameda Prof. Hernani Monteiro, 4200-319 Porto, Portugal

**Keywords:** Intracerebral hemorrhage, Prognosis, Clinical prediction rules, Mortality, Morbidity

## Abstract

**Background:**

Prognostic tools for intracerebral hemorrhage (ICH) patients are potentially useful for ascertaining prognosis and recommended in guidelines to facilitate streamline assessment and communication between providers. In this systematic review with meta-analysis we identified and characterized all existing prognostic tools for this population, performed a methodological evaluation of the conducting and reporting of such studies and compared different methods of prognostic tool derivation in terms of discrimination for mortality and functional outcome prediction.

**Methods:**

PubMed, ISI, Scopus and CENTRAL were searched up to 15th September 2016, with additional studies identified using reference check. Two reviewers independently extracted data regarding the population studied, process of tool derivation, included predictors and discrimination (c statistic) using a predesignated spreadsheet based in the CHARMS checklist. Disagreements were solved by consensus. C statistics were pooled using robust variance estimation and meta-regression was applied for group comparisons using random effect models.

**Results:**

Fifty nine studies were retrieved, including 48,133 patients and reporting on the derivation of 72 prognostic tools. Data on discrimination (c statistic) was available for 53 tools, 38 focusing on mortality and 15 focusing on functional outcome. Discrimination was high for both outcomes, with a pooled c statistic of 0.88 for mortality and 0.87 for functional outcome. Forty three tools were regression based and nine tools were derived using machine learning algorithms, with no differences found between the two methods in terms of discrimination (*p* = 0.490). Several methodological issues however were identified, relating to handling of missing data, low number of events per variable, insufficient length of follow-up, absence of blinding, infrequent use of internal validation, and underreporting of important model performance measures.

**Conclusions:**

Prognostic tools for ICH discriminated well for mortality and functional outcome in derivation studies but methodological issues require confirmation of these findings in validation studies. Logistic regression based risk scores are particularly promising given their good performance and ease of application.

**Electronic supplementary material:**

The online version of this article (10.1186/s12874-018-0613-8) contains supplementary material, which is available to authorized users.

## Background

Intracerebral hemorrhage (ICH) is a major cause of death and disability, with an incidence rate of 24.6 per 100,000 person-years and a fatality rate of 40%. After such event, only 12–39% of patients regain independence [1]. Contrary to ischemic stroke, medical care for ICH remains mostly supportive, and few interventions clearly demonstrated benefit in this population [2, 3]. Several prognostic tools have been proposed for mortality and functional outcome prediction in ICH. These tools are potentially useful for ascertaining prognosis, facilitating communication between clinicians, characterizing and selecting patients for interventions, and for benchmarking purposes in healthcare delivery [2, 4].

The aim of this study was to systematically identify, assess and review the methodological conduct and reporting of studies deriving prognostic tools for the risk of death and/or functional recovery after ICH and to evaluate their overall discrimination according to the method of derivation and type of outcome.

## Methods

We have designed, developed and reported our systematic review and meta-analysis in accordance with recommendations from the Cochrane Prognosis Methods Group [5] and the PRISMA [6] and MOOSE [7] guidelines. For this purpose, we searched PubMed, ISI Web of Knowledge, Scopus, and CENTRAL for all studies reporting the derivation of prognostic tools for predicting death and/or functional recovery after non-traumatic ICH, using the broad and sensitive search query reported Additional file 1. The search included articles from database inception to 15th September 2016, with additional articles identified from reference checking. No language restrictions were applied. There is no protocol available.

### Study selection and inclusion criteria

Articles were included if they met the following criteria: 1) were human studies; 2) were original articles; 3) were adult studies (≥ 18 years); 4) did not consist of case reports/ case series; 5) enrolled non-traumatic ICH patients; 6) were prognostic studies; 7) described the application of a prognostic tool; and 8) were derivation studies. Studies involving traumatic and/or extra-axial bleedings were excluded. Study selection was performed using a two-step process. In the first step (screening), all abstracts were reviewed by two authors independently applying the inclusion criteria. This process was repeated in the second step again by two authors working independently, applying the same criteria to the full text of remaining studies. Disagreements were resolved by consensus.

### Quality assessment, data extraction, analysis and reporting

To inform quality assessment and data extraction from individual studies, two reviewers independently applied a spreadsheet based in the CHARMS checklist [5] to the included studies, gathering information on the following aspects of prognostic tool derivation: 1) population, sampling and source of data; 2) outcome timing and definition; 3) number and type of predictors; 4) number of patients and events 5) handling of missing data; 6) method for tool derivation and 7) prognostic tool performance.

Prognostic tool performance was evaluated by determining its discriminatory capacity, i.e., its ability to determine which patients will suffer the outcome of interest. As a measure of this, we retrieved the c-statistic along with its 95% confidence interval (CI). For studies not reporting any of these parameters, we obtained them by recreating the receiver operating characteristic (ROC) curve from reported probability distributions; for studies reporting the c-statistic but not its confidence interval, we calculated the later using the method reported by Hanley and McNeil [8], where the number of outcomes was available. Standard errors were derived from the respective CIs.

Given the fact that some authors derived more than one tool from the same sample population, we pooled c-statistics using robust variance estimation (RVE) to account for dependent effects, according to Tanner-Smith et al. [9]. Specifically, we assumed correlated effect sizes and used a random effects model with inverse variance weights to estimate the overall mean c-statistic and mean c-statistics for mortality prediction tools, functional outcome prediction tools, logistic regression based tools, and machine learning algorithms. Univariate meta-regression was used to compare these groups and *p* values < 0.05 were considered significant. Due to the nature of the meta-analytical technique used, heterogeneity statistics such as Q-statistic and I-square are not recommended, according to Tanner-Smith et al. [9]. However, the I2 statistic is reported for illustrative purposes. Statistical analysis was performed using specific macros [9] designed for R and SPSS® statistics v 24.0.

## Results

**Fig. 1:**
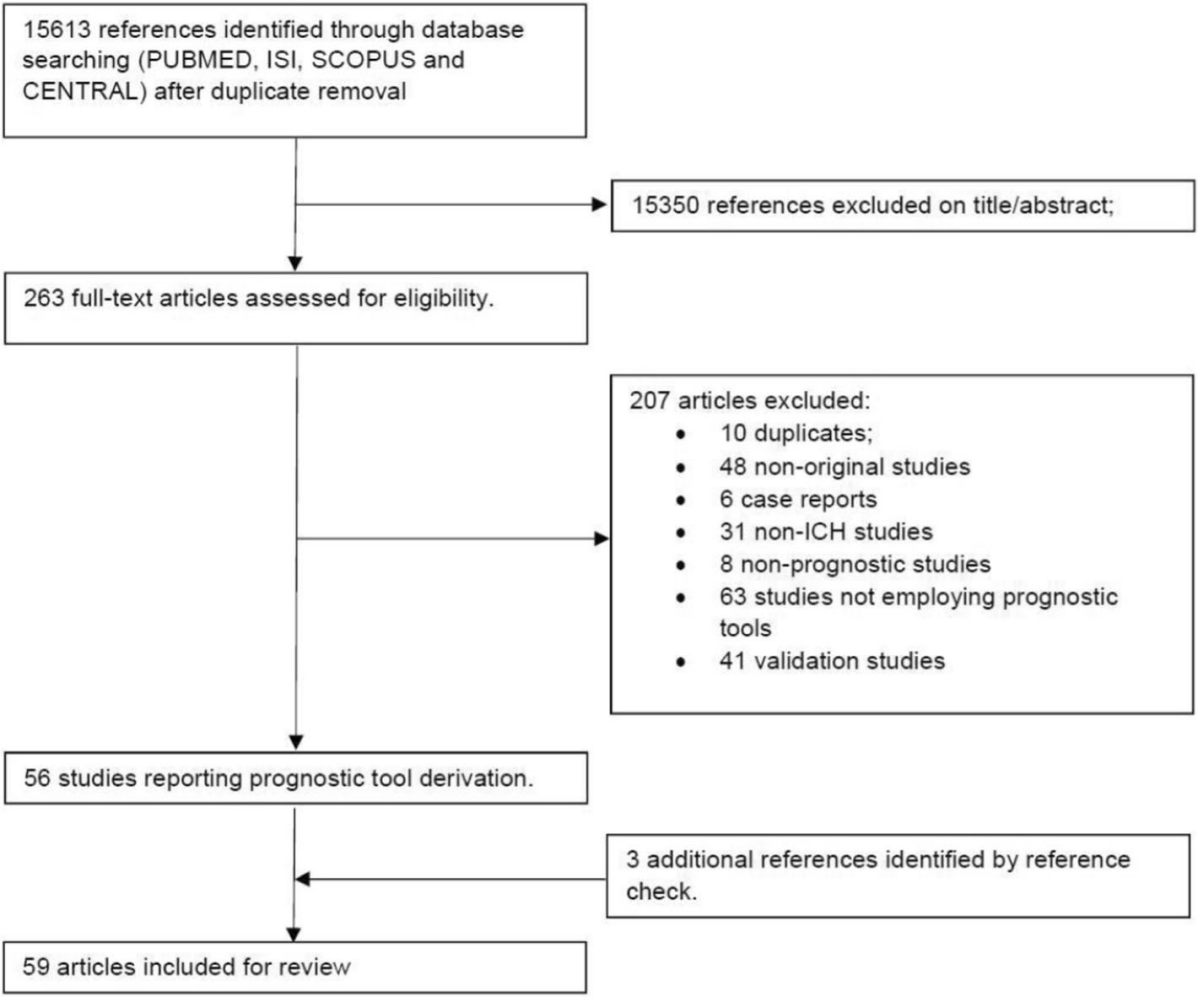
Study selection flow chart

Figure 1 depicts the study selection procedure. The search query retrieved 15,613 references: after the screening step, there were 263 references left for full text review. The second step removed an additional 207 references, leaving us with 56 studies reporting the derivation of at least one prognostic tool. Three additional studies were identified through reference check, which led to the final number of 59 studies involving 48,133 patients. Nine studies reported the derivation of more than one prognostic tool, so the total number of prognostic tools analyzed was 72. The summary description of these tools is depicted in Table 1.

**Table 1:** Summary description of prognostic tools

Author	Year	Population	Tool	Timing	Variables	AUC (SE)
Mortality prediction tools						
Alsina [24]	2014	Supratentorial ICH not submitted to surgery	Equation	30 days	IVH, hematoma size, and midline shift.	0·933 (0·029)
Berwaerts [41]	2000	Oral anticoagulant related ICH	Equation	Discharge	Hematoma diameter and CT signs of ischemia.	–
Bhatia [65]	2013	Primary ICH	Equation	Discharge	GCS, hematoma size, IVH,and ventilatory requirement.	0·822 (0·033)
Broderick [44]	1993	Spontaneous ICH	Equation	30 days	GCS, hematoma size.	0·805 (0·036)
Broderick´ [44]	1993	Spontaneous ICH	Equation	30 days	Hematoma size, IVH volume, GCS, and surgery.	–
Celik [68]	2014	Spontaneous ICH	ANN	10 days	Age, gender, hypertension, diabetes, smoking, mean blood pressure, Scandinavian Stroke Scale score, pulse pressure, localization of hemorrhage (including infratentorial), volume of hemorrhage, ventricular drainage, and midline shift.	–
Cerillo [21]	1981	Operated supratentorial ICH	Equation	Discharge	Age, mode of onset, site of hemorrhage, level of consciousness, time from onset to surgery, congestive heart failure/coronary artery disease, and diabetes/uremia.	0·893 (0·033)
Chen [45]	2011	Nontraumatic ICH	Score	Discharge	GCS, hematoma volume, IVH, and diabetes.	0·867 (0·027)
Chiu [61]	2016	Spontaneous ICH	CART+SVM	30 days	GCS, hematoma size.	–
Chuang [63]	2009	Spontaneous ICH	Score	30 days	Age, GCS, hypertension, glucose and dialysis dependency.	0·890 (0·026)
Edwards [28]	1999	Supratentorial ICH	ANN	Discharge	Gender, race, hydrocephalus, mean arterial pressure, pulse pressure, GCS, IVH, hematoma size, location (thalamic, basal, lobal), cisternal effacement, pineal shift, hypertension, diabetes, and age.	0·984 (0·020)
Edwards´ [28]	1999	Supratentorial ICH	Equation	Discharge	Hydrocephalus, GCS, gender, pineal shift	0·919 (0·043)
Fogelholm [31]	1997	Supratentorial ICH	Equation	28 days	Consciousness, mean arterial pressure, subarachnoid spread, midline shift, glucose, and vomiting.	–
Frithz [12]	1976	ICH patients < 70 years	Decision tree	Discharge	Consciousness, diastolic blood pressure.	0·943 (0·024)
Galbois [38]	2013	Spontaneous comatose ICH not submitted to surgery	Score	ICU stay	Brainstem reflexes, swirl sign.	0·850 (0·050)
Galbois´ [38]	2013	Spontaneous comatose ICH not submitted to surgery	Score	ICU stay	Corneal reflexes, swirl sign.	0·840 (0·051)
Grellier [66]	1983	Spontaneous ICH	Score	2 days	Age, gender, consciousness (normal, changed, coma), CV risk factors (alcohol, tobacco, hypertension, dyslipidemia, CV disease), and ICH location (infratentorial, thalamic, internal capsule, oval center, lobar).	–
Hallevi [34]	2009	Primary ICH with IVH	Score	Discharge	GCS, total volume (ICH + IVH).	0·840^a^
Hemphill [48]	2001	Nontraumatic ICH	Score	30 days	Age, ICH volume, infratentorial ICH, GCS, and IVH.	0·920 (0·020)
Ho [64]	2016	Primary ICH	Score	Discharge	Age, creatinine, NIHSS, heart disease, gender, and systolic blood pressure.	0·870 (0·018)
Huang [40]	2008	Spontaneous medically treated ICH in hemodialysis patients	Score	30 days	GCS, age, and systolic blood pressure.	0·745 (0·048)
Li [50]	2012	Spontaneous ICH	Equation	Discharge	Age, GCS, glucose, and white blood cell count.	0·923 (0·020)
Li´ [49]	2011	Primary ICH	Score	Discharge	Age, Glucose, LDH, and white blood cell count.	0·745 (0·025)
Lukic [33]	2012	Primary supratentorial medically treated ICH	Equation	Discharge	Level of consciousness, GCS verbal response, age, gender, and pulse pressure.	0·856 (0·018)
Lukic´ [26]	2012	Spontaneous supratentorial ICH	ANN	Discharge	Age, gender, pulse pressure, mean arterial pressure, GCS (E/V/M), and consciousness.	0·883 (0.048)
Lukic´´ [26]	2012	Spontaneous supratentorial ICH	Equation	Discharge	GCS, level of consciousness.	0·819 (0·030)
Masé [27]	1995	Primary supratentorial medically treated ICH	Equation	30 days	GCS, IVH spread, and hematoma size.	–
Parry-Jones [53]	2013	Spontaneous ICH	Equation	30 days	Age, GCS, IVH extension, and hematoma volume.	0·897 (0·010)
Peng [54]	2010	Spontaneous ICH	Random Forrest	30 days	Age, gender, hypertension, diabetes, ischemic heart disease, previous stroke, anemia, dialysis dependency, GCS, systolic/diastolic/mean blood pressure, infratentorial bleed, site of ICH, ICH volume, IVH, pineal shift, hydrocephalus, hemoglobin, and glucose.	0·870 (0·015)
Peng´ [54]	2010	Spontaneous ICH	ANN	30 days	Age, gender, GCS, site of ICH, ICH volume, IVH, hypertension, diabetes, anemia, and previous stroke.	0·810 (0·020)
Peng´´ [54]	2010	Spontaneous ICH	SVM	30 days	Age, gender, GCS, site, ICH volume, IVH, hypertension, diabetes, anemia, and previous stroke.	0·790 (0·020)
Peng´´´ [54]	2010	Spontaneous ICH	Equation	30 days	Anemia, age, GCS, hypertension, and dialysis dependency.	0·780 (0·020)
Romano [56]	2009	Primary ICH	Score	30 days	GCS, hematoma volume, and intraventricular spread.	0·915 (0·026)
Ruiz-Sandoval [58]	2007	Primary ICH	Score	Discharge	Age, infratentorial bleed, ICH size, GCS, and IVH spread.	0·880 (0·017)
Safatli [60]	2016	Primary ICH	Score	30 days	GCS, infratentorial bleed, and hematoma volume.	–
Szepesi [32]	2015	Supratentorial ICH	Equation	30 days	Age, hematoma volume, IVH, systolic blood pressure, glucose, and potassium.	–
Tabak [59]	2007	Spontaneous ICH	Equation	Discharge	Age, creatinine, glucose, pH, CO2, O2, partial thromboplastin time, prothrombin time, platelets, white blood cells, cancer, temperature, pulse, systolic blood pressure, respiratory rate, and altered mental status.	0·890 (0·003)
Takahashi [67]	2006	Spontaneous ICH	CART	Discharge	Japan Coma Scale, ICH volume, and age.	0·853 (0·024)
Takahashi´ [67]	2006	Spontaneous ICH	Equation	Discharge	Japan Coma Scale, temperature, infratentorial bleed, and ICH volume.	0·810 (0·033)
Tshikwela [36]	2012	Black hypertensive primary ICH	Score	Discharge	GCS, ICH volume, left hemisphere involved.	–
Tshikwela´ [36]	2012	Black hypertensive primary ICH	Score	Discharge	Gender, GCS, midline shift.	–
Tuhrim [23]	1999	Primary supratentorial ICH managed medically	Equation	30 days	GCS, ICH volume, pulse pressure, hydrocephalus, and IVH volume.	–
Tuhrim´ [30]	1991	Supratentorial ICH	Equation	30 days	Hematoma size, IVH, GCS, pulse pressure, and IVH^a^GCS interaction.	0·900 (0·027)
Tuhrim´´ [29]	1988	Supratentorial hemorrhage	Equation	30 days	GCS score, hematoma size, and pulse pressure.	0·892 (0·042)
Ziai [35]	2015	Primary ICH with IVH	Score	Discharge	Temperature, glucose, intracranial pressure, and Do-Not-Resuscitate orders	0·850 (0·030)
Zis [39]	2014	Non-operated primary ICH	Score	30 days	GCS, ICH size, INR, IVH spread, and infratentorial location.	0·920 (0·023)
Functional outcome prediction tools						
Appelboom [10]	2012	AVM related ICH	Score	3 months	Age, IVH, infratentorial bleed, GCS, and hematoma size.	0·914 (0·039)
Creutzfeld [47]	2011	Primary ICH	Equation	Discharge	Age, GCS, heart rate, mass effect, IVH, premorbid level of function, and systolic blood pressure.	0·930 (0·014)
Flemming [18]	2001	Lobar primary supratentorial ICH	Tree based model	Discharge	GCS, septum pellucidum shift.	0·890 (0·045)
Flemming´ [18]	2001	Lobar primary supratentorial ICH	Tree based model	Discharge	ICH size, GCS, and time to presentation.	0·921 (0·032)
Hallevy [25]	2002	Primary supratentorial medically treated ICH	Score	Discharge	Age, limb paresis, level of consciousness, mass effect, hematoma size, and intraventricular extension.	0·897 (0·023)
Ji [51]	2013	Spontaneous ICH	Score	1 year	Age, NIHSS, GCS, glucose, infratentorial bleed, ICH volume, and IVH.	0·836 (0·009)
Lisk [22]	1994	Primary supratentorial < 24 h	Equation	Discharge or 30 days	Age, GCS, hemorrhage volume, and gender.	–
Lisk´ [22]	1994	Primary supratentorial < 24 h, GCS > 9, no surgery	Equation	Discharge or 30 days	Age, hemorrhage diameter, and ventricular extension.	–
Neidert [11]	2016	AVM related ICH	Score	Unclear	Age, GCS, hematoma size, IVH, AVM size, diffuse nidus, eloquence, and deep venous drainage.	0·842 (0·046)
Misra [15]	1999	Primary putaminal ICH	Equation	3 months	GCS, pupillary change, incontinence, and location of hematoma (cortical, subcortical, medial or lateral).	–
Mittal [52]	2011	Primary ICH	Score	Discharge	Age, infratentorial, ICH size, GCS, cognitive impairment, and FOUR score.	–
Portenoy [20]	1987	Nontraumatic supratentorial spontaneous ICH	Equation	Unclear	GCS, ICH size (index), and IVH spread.	–
Poungvarin [55]	2006	Primary ICH	Equation	Discharge	Fever, ICH size > 30, GCS, and IVH spread.	–
Rost [57]	2008	Primary ICH	Score	3 months	Age, GCS, hematoma size, location (infratentorial/deep/lobar), and cognitive impairment.	0·879 (0·017)
Shah [17]	2005	Thalamic hemorrhage	Equation	3 months	Posterolateral ICH extension, Canadian Neurological Scale.	–
Shaya [19]	2005	Hypertensive supratentorial ICH	Score	6 months	Focal neurological deficit, hydrocephalus, ICH volume	–
Weimar [42]	2009	Patients included in ICH trials	Equation	3 months	Age, NIHSS, and level of consciousness.	0·805 (0·020)
Weimar´ [37]	2006	Non-comatose ICH patients	Equation	100 days	Age, NIHSS.	0·861 (0·029)
Weimar´´ [43]	2006	Spontaneous ICH	Score	100 days	Age, NIHSS, and level of consciousness.	0·913 (0·018)
Combined outcome prediction tools						
Cheung [46]	2003	Nontraumatic ICH	Score	30 days	IVH, subarachnoid extension, pulse pressure, NIHSS, and temperature.	–
						–
Cheung´ [46]	2003	Nontraumatic ICH	Score	30 days	Age, IVH, infratentorial bleed, NIHSS, and hematoma size.	–
						–
Cho [14]	2008	Basal ganglia hemorrhage	Score	6 months	GCS, ICH volume, and IVH.	0·897 (0·033)^b^
						Barthel 0·884^a^ GOS 0·935^ac^
Godoy [62]	2006	Primary ICH	Score	30 days^b^	Age, GCS, Graeb score, ICH volume, and APACHE2 score comorbidities.	0·878 (0·028)^b^
				6 months^c^		0.893 (0·025)^c^
Godoy´ [62]	2006	Primary ICH	Score	30 days^b^	Age, GCS, Graeb score, ICH volume, and APACHE2 score comorbidities.	0·869 (0·029)^b^
				6 months^c^		0·895 (0·024)^c^
Lei [13]	2016	Cerebral amyloid related ICH	Score	3 months	Age, IVH, midline shift, and GCS.	0·890 (0·038)^b^
						0·810 (0·031)^c^
Stein [16]	2010	Supratentorial deep ICH with secondary IVH	Score	30 days^b^	Age, GCS, hydrocephalus, and ICH volume	0·890 (0.036)^b^
				6 months^c^		0·848 (0·056)^c^

*SE* standard error, *ICH* intracerebral hemorrhage, *IVH* intraventricular hemorrhage, *CT* computerized tomography, *GCS* Glasgow Coma Scale, *ANN* artificial neural networks, *CART* classification and regression tree, *SVM* support vector machine, *ICU* intensive care unit, *CV* cardiovascular, *NIHSS* National Institute of Health Stroke Scale, *LDH* lactate dehydrogenase, *INR* International normalized ratio, *AVM* arteriovenous malformation, *GOS* Glasgow Outcome Score

^a^C-statistics were reported but standard errors were not reported, nor were the number of outcomes

^b^Mortality

^c^Functional outcome

### Population, sampling and source of data

The source population from which the patients were recruited for prognostic tool derivation consisted on primary/spontaneous ICH patients for all tools except two [10, 11], which focused on arteriovenous malformation related hemorrhages (Table 1). However, several studies included further specifications for patient inclusion namely age [12], cerebral amyloid related angiopathy [13], deep location [14–17], lobar location [18], supratentorial bleeds [16, 19–33], presence of intraventricular hemorrhage [16, 34, 35], African ethnicity [36], non-comatose patients [22, 37], comatose patients [38], medically treated patients [22–25, 27, 33, 38–40], surgically treated patients [21], oral anticoagulant related bleeds [41], hypertensive patients [19, 36], and dialysis patients [40]. The majority of studies (*n* = 40) recruited patients from hospitals or emergency rooms [10–14, 16–22, 26, 29–31, 36, 40–61] but nine studies recruited patients from intensive care units [24, 28, 32, 33, 35, 38, 62–64], three studies recruited patients from stroke units [34, 37, 65], six studies recruited patients from neurology/neurosurgery departments [15, 25, 27, 39, 66, 67], and one study recruited patients from both an intensive care unit and a stroke unit [23]. Most prognostic tools were derived from cohort studies, with the exceptions being registries [29, 30, 43, 51, 64], randomized clinical trial data [14, 42], case-control studies [26], and administrative databases [59]. Thirteen studies were multicentric [13, 24, 29, 30, 35–37, 42, 43, 51, 55, 59, 62], with two studies involving more than two countries [42, 55]. The sampling method was not reported or unclear for 18 studies [12, 15, 17, 21, 23, 24, 26, 29, 30, 32, 36, 42, 54, 61, 63, 66–68], being consecutive for all others.

### Outcome timing, definition and assessment

Of the 72 prognostic tools included in this review 46 focused on mortality [12, 21, 23, 24, 26–36, 38–41, 44, 45, 48–50, 53, 54, 56, 58–61, 63–68], 19 focused on morbidity [10, 11, 15, 17–20, 22, 25, 37, 42, 43, 47, 51, 52, 55, 57], and seven were derived for a combined outcome (mortality plus morbidity) [13, 14, 16, 46, 62].

Mortality prediction was mostly attempted at hospital discharge or 1 month (Table 1); exceptions to this rule were the studies by Grellier [66], Celik [68], Lei [13], Cho [14], and Galbois [38], which analyzed death at 2 days, 10 days, 3 months, 6 months, and ICU discharge respectively. Interestingly, Galbois focused on brain death rather than the general concept of mortality used in other studies. Functional status prediction was more heterogeneous on the timing and method of assessment: ten tools attempted to predict functional status at discharge/ 1 month [18, 22, 25, 46, 47, 52, 55], eight tools attempted to predict at 3 months [10, 13, 15, 17, 37, 42, 43, 57], five tools attempted to predict at 6 months [14, 16, 19, 62], and one tool attempted to predict at 1 year [51]. The studies by Portenoy [20] and Neidert [11] were unclear about the time of outcome assessment. The instrument for functional outcome evaluation also differed between studies: ten studies used the modified Rankin scale [10, 11, 13, 16, 22, 25, 46, 47, 51, 55], six studies used the Glasgow Outcome scale [14, 18, 19, 52, 57, 62], six studies used the Barthel index [14, 15, 17, 37, 42, 43], and one study used a subjective assessment [20]. Only six studies reported blinded outcome assessment [10, 13, 37, 43, 51, 52]. All outcomes were binary except in the study by Shaya [19], where the outcome was ordinal.

### Number and type of predictors

**Fig. 2:**
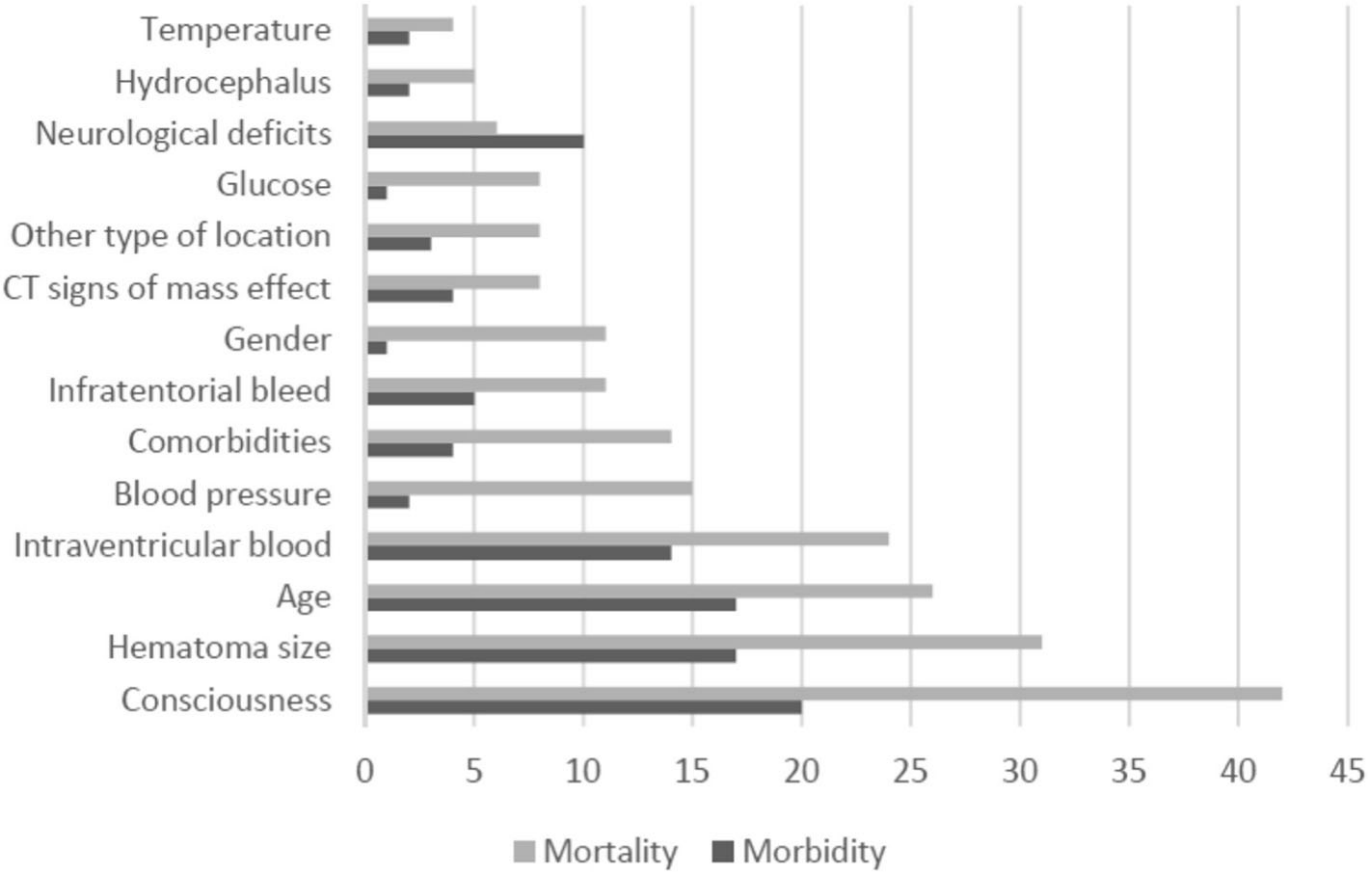
Predictor distribution according to mortality vs functional outcome prediction tool

The number of predictors for each prognostic tool ranged from two to 20, with the mode being three (Table 2). The five most frequently included predictors were consciousness (*n* = 57), hematoma size (*n* = 43), age (*n* = 38), intraventricular blood (*n* = 32), and the presence of comorbidities (*n* = 16). Figure 2 stratifies the ten most frequently used variables for mortality and functional outcome prediction.

**Table 2:** summary description of the tool development process and risk of bias

Author	Source of data	Sampling reported	Nr patients	Nr events	Nr variable	EPV	Loss to follow-up:	Missing data reported?	Blinding reported?	Modelling method	Internal validation	Calibration
Alsina [24]	Cohort	Not reported	100	38	3	12.7	0%	Yes	No	Logistic	No	Hosmer-Lemeshow
Berwaerts [41]	Cohort	Consecutive	42	18	2	9	0%	Yes	No	Logistic	No	Not reported
Bhatia [65]	Cohort	Consecutive	214	70	4	17.5	0%	No	No	Logistic	No	Not reported
Broderick [44]	Cohort	Consecutive	162	83	2	19.8	0.6%	Yes	No	Logistic	No	Not reported
Broderick´ [44]	Cohort	Consecutive	162	83	4	39.5	0.6%	Yes	No	Logistic	No	Not reported
Celik [68]	Cohort	Not reported	257	119	12	9.9	0%	No	No	ANN	Cross-validation	Not reported
Cerillo [21]	Cohort	Not reported	88	34	7	4.9	0%	No	No	Univariate analysis	No	Not reported
Chen [45]	Cohort	Consecutive	285	61	4	15.3	0%	No	No	Logistic	No	Not reported
Chiu [61]	Cohort	Not reported	106	16	2	8	0%	Yes	No	CART + SVM	Split sample	Not reported
Chuang [63]	Cohort	Not reported	293	40	5	8	0%	No	No	Logistic	Cross-validation	Hosmer-Lemeshow
Edwards [28]	Cohort	Consecutive	81	21	15	1.4	0%	No	No	ANN	No	Hosmer-Lemeshow
Edwards´ [28]	Cohort	Consecutive	81	21	4	5.3	0%	Yes	No	Logistic	No	Hosmer-Lemeshow
Fogelholm [31]	Cohort	Consecutive	282	120	6	20	0%	Yes	No	Logistic	No	Not reported
Frithz [12]	Cohort	Not reported	91	79	2	6	0%	Yes	No	CART	No	Not reported
Galbois [38]	Cohort	Consecutive	72	35	2	17.5	0%	Yes	No	Logistic	Cross-validation	Not reported
Galbois´ [38]	Cohort	Consecutive	72	35	2	17.5	0%	Yes	No	Logistic	Cross-validation	Not reported
Grellier [66]	Cohort	Not reported	300	Not reported	9	n/a	0%	No	No	Unclear	No	Not reported
Hallevi [34]	Cohort	Consecutive	174	Not reported	2	n/a	0%	Yes	No	Logistic	No	Not reported
Hemphill [48]	Cohort	Consecutive	152	68	5	13.6	0%	Yes	No	Logistic	No	Not reported
Ho [64]	Registry	Consecutive	805	164	6	27.3	0%	No	No	Logistic	No	Le Cessie and Howelingen + plots
Huang [40]	Cohort	Consecutive	107	72	3	11.7	0%	Yes	No	Logistic	No	Not reported
Li [50]	Cohort	Consecutive	227	49	4	12.3	0%	Yes	No	Logistic	No	Not reported
Li´ [49]	Cohort	Consecutive	716	140	4	35	0%	Yes	No	Logistic	No	Not reported
Lukic [33]	Cohort	Consecutive	411	256	5	31	0%	Yes	No	Logistic	No	Hosmer-Lemeshow
Lukic´ [26]	Case-Control	Not reported	200	100	8	12.5	0%	Yes	No	ANN	Split Sample	Not reported
Lukic´´ [26]	Case-Control	Not reported	200	100	2	50	0%	Yes	No	Logistic	No	Not reported
Masé [27]	Cohort	Consecutive	138	38	3	12.7	0%	No	No	Logistic	No	Not reported
Parry-Jones [53]	Cohort	Consecutive	1175	483	4	120.8	1.1%	Yes	No	Logistic	No	Not reported
Peng [54]	Cohort	Not reported	423	62	20	3.1	0%	Yes	No	Random Forrest	Cross-validation	Not reported
Peng´ [54]	Cohort	Not reported	423	62	10	6.2	0%	Yes	No	ANN	Cross-validation	Not reported
Peng´´ [54]	Cohort	Not reported	423	62	10	12.4	0%	Yes	No	SVM	Cross-validation	Not reported
Peng´´´ [54]	Cohort	Not reported	423	62	5	12.4	0%	Yes	No	Logistic	Cross-validation	Not reported
Romano [56]	Cohort	Consecutive	154	63	3	21	0.6%	Yes	No	Logistic	Split sample	Not reported
Ruiz-Sandoval [58]	Cohort	Consecutive	378	174	5	34.8	0%	Yes	No	Logistic	Bootstrap	Hosmer-Lemeshow
Safatli [60]	Cohort	Consecutive	342	86	3	28.7	0%	No	No	Logistic	No	Hosmer-Lemeshow
Szepesi [32]	Cohort	Not reported	125	59	6	9.8	0%	Yes	No	Logistic	No	Hosmer-Lemeshow
Tabak [59]	Administrative data	Consecutive	29,975	6765	17	397.9	0%	Yes	No	Logistic	Bootstrap	Calibration plot
Takahashi [67]	Cohort	Not reported	347	70	3	23.3	0%	No	No	CART	Cross-validation	Not reported
Takahashi´ [67]	Cohort	Not reported	347	70	4	17.5	0%	No	No	Logistic	No	Not reported
Tshikwela [36]	Cohort	Not reported	185	68	3	22.7	0%	No	No	Logistic	No	Not reported
Tshikwela´ [36]	Cohort	Not reported	185	68	3	22.7	0%	No	No	Logistic	No	Not reported
Tuhrim [23]	Cohort	Not reported	129	27	5	5.4	0%	No	No	Logistic	No	Not reported
Tuhrim´ [30]	Registry	Not reported	187	54	5	10.8	2.1%	Yes	No	Logistic	No	Not reported
Tuhrim´´ [29]	Registry	Not reported	73	25	3	8.3	0%	Yes	No	Logistic	No	Not reported
Ziai [35]	Cohort	Consecutive	170	87	4	20.8	0%	Yes	No	Logistic	Cross-validation	Not reported
Zis [39]	Cohort	Consecutive	191	61	5	12.2	0%	No	No	Logistic	No	Hosmer-Lemeshow
Appelboom [10]	Cohort	Consecutive	84	18	5	3.6	Unclear	Yes	Yes	Logistic (Update)	No	Not reported
Creutzfeld [47]	Cohort	Consecutive	424	187	7	26.7	0%	No	No	Logistic	No	Hosmer-Lemeshow
Flemming [18]	Cohort	Consecutive	81	24	2	12	0%	Yes	No	Decision Tree	No	Not reported
Flemming´ [18]	Cohort	Consecutive	81	51	3	10	0%	Yes	No	Decision Tree	No	Not reported
Hallevy [25]	Cohort	Consecutive	184	70	6	11.7	0%	No	No	Logistic	No	Not reported
Ji [51]	Registry	Consecutive	1953	912	7	130.3	12.6%	Yes	Yes	Logistic	Split sample	Hosmer-Lemeshow
Lisk [22]	Cohort	Consecutive	75	35	4	8.8	0%	Yes	No	Logistic	No	Hosmer-Lemeshow
Lisk´ [22]	Cohort	Consecutive	42	9	3	3	0%	Yes	No	Logistic	No	Hosmer-Lemeshow
Neidert [11]	Cohort	Consecutive	67	28	8	3.5	0%	No	No	Univariate analysis	No	Not reported
Misra [15]	Cohort	Not reported	38	Not reported	4	n/a	Unclear	Yes	No	Logistic	No	Not reported
Mittal [52]	Cohort	Consecutive	92	62	5	6	0%	No	Yes	Logistic (update)	No	Not reported
Portenoy [20]	Cohort	Consecutive	112	41	3	13.7	0%	No	No	Logistic	No	Hosmer-Lemeshow
Poungvarin [55]	Cohort	Consecutive	995	402	4	100.5	0%	Yes	No	Logistic	No	Not reported
Rost [57]	Cohort	Consecutive	418	121	5	24.2	13.4%	Yes	No	Logistic	Split sample	Not reported
Shah [17]	Cohort	Not reported	53	29	2	12	0%	No	No	Logistic	No	Not reported
Shaya [19]	Cohort	Consecutive	50	n/a	3	n/a	0%	No	No	Ordered logistic	No	Not reported
Weimar [42]	RCTs	Not reported	564	171	3	57	0%	Yes	No	Logistic (update)	No	Calibration plot
Weimar´ [37]	Cohort	Consecutive	207	78	2	39	20.4%	Yes	Yes	Logistic	No	Not reported
Weimar´´ [43]	Registry	Consecutive	340	89	3	29.7	27%	Yes	Yes	Logistic (update)	No	Not reported
Cheung [46]	Cohort	Consecutive	141	31^a^	5	6.2^a^	0.7%	Yes	No	Logistic	No	Not reported
				49^b^		9.8^b^						
Cheung´ [46]	Cohort	Consecutive	141	31^a^	5	6.2^a^	0.7%	Yes	No	Logistic (update)	No	Not reported
				49^b^		9.8^b^						
Cho [14]	RCT	Consecutive	226	42^a^	3	14^a^	0%	Yes	No	Logistic	No	Not reported
				Unclear^b^		n/a^b^						
Godoy [62]	Cohort	Consecutive	153	53^a^	5	10.6^a^	0%	Yes	No	Logistic (update)	No	Not reported
				59^b^		11.8^b^						
Godoy´ [62]	Cohort	Consecutive	153	53^a^	5	10.6^a^	0%	Yes	No	Logistic (update)	No	Not reported
				59^b^		11.8^b^						
Lei [13]	Cohort	Consecutive	170	43^a^	4	10.8^a^	0%	No	Yes	Logistic	Split sample	Not reported
				90^b^		20^b^						
Stein [16]	Cohort	Consecutive	110	31^a^	4	7.8^a^	0%	Yes	No	Logistic	Split sample	Not reported
				86^b^		4.5^b^						

*ANN* artificial neural networks, *CART* classification and regression tree, *SVM* support vector machine

^a^Values relating to mortality

^b^Values relating to functional outcome

### Number of patients and events

The number of included patients varied between 38 [15] and 29,775 [59] and the number of outcomes ranged from 9 [22] to 6765 [59] (Table 2), with four studies not reporting this item [14, 15, 34, 66]. The event per variable (EPV) rate ranged from 1.4 [28] to 398 [59], with 21 derivations showing a rate < 10 [10–12, 16, 21–23, 28, 29, 32, 41, 46, 52, 54, 61, 63, 68].

### Handling of missing data and loss to follow-up

Handling of missing data was not reported or unclear in 22 studies [11, 13, 17, 19–21, 23, 25, 27, 28, 36, 39, 45, 47, 52, 60, 63–68] (Table 2). Among studies reporting this item, all of them except two used a complete case analysis, with the exceptions using a missing cathegory [37, 59]. Two studies failed to report the number of patients lost to follow-up [10, 15]: as for the others, the majority of them showed a 100% complete follow-up but five studies showed a loss < 5% [30, 44, 46, 53, 56], two studies showed a loss of 5–20% [51, 57] and two studies showed a loss > 20% [37, 43].

### Methods used for tool derivation

Amongst the 72 prognostic tools encountered, 58 were regression based [10, 13–17, 19, 20, 22–60, 62–65, 67], 11 were machine learning algorithms [12, 18, 26, 28, 54, 61, 67, 68], two were based on univariate analysis [11, 21], and one was unclear on the method of derivation [66] (Table 2). Within the regression based tools, 51 were newly derived [13–17, 19, 20, 22–41, 44–51, 53–60, 63–65, 67] and seven were model updates [10, 42, 43, 46, 52, 62]. Newly derived models preferentially used automated methods (forward/backward stepwise) for predictor selection during multivariate modelling (reported in 30 derivation procedures [14–17, 19, 20, 22, 23, 25, 26, 28–31, 33, 35–37, 40, 41, 44, 46, 48, 49, 51, 54, 56, 63–65]), whereas the method was unclear for ten [13, 24, 32, 38, 45, 55, 57, 58, 60]. Model updates consisted in intercept recalibrations [42], modifications of cut-off levels for specific variables [10], and removal or introduction of new variables [43, 46, 52, 62]. Of the 58 regression based tools found, more than half (32) were presented as scores [10, 11, 13, 14, 16, 19, 25, 34–36, 38–40, 43, 45, 46, 48, 49, 51, 52, 56–58, 60, 62–64, 66]. Machine learning methods employed were artificial neural networks used in four tools [26, 28, 54, 68], decision trees used in four tools [12, 18, 67], support vector machine used in one tool [54], random forests used in one tool [54], and a hybrid approach (decision tree + support vector machine) also used in one tool [61] (Table 1). Internal validation methods were used in 19 derivations: bootstrapping was used in two [58, 59], cross-validation was used in ten [35, 38, 54, 63, 67, 68] and split sample was used in seven [13, 16, 26, 51, 56, 57, 61].

### Prognostic tool performance

**Fig. 3:**
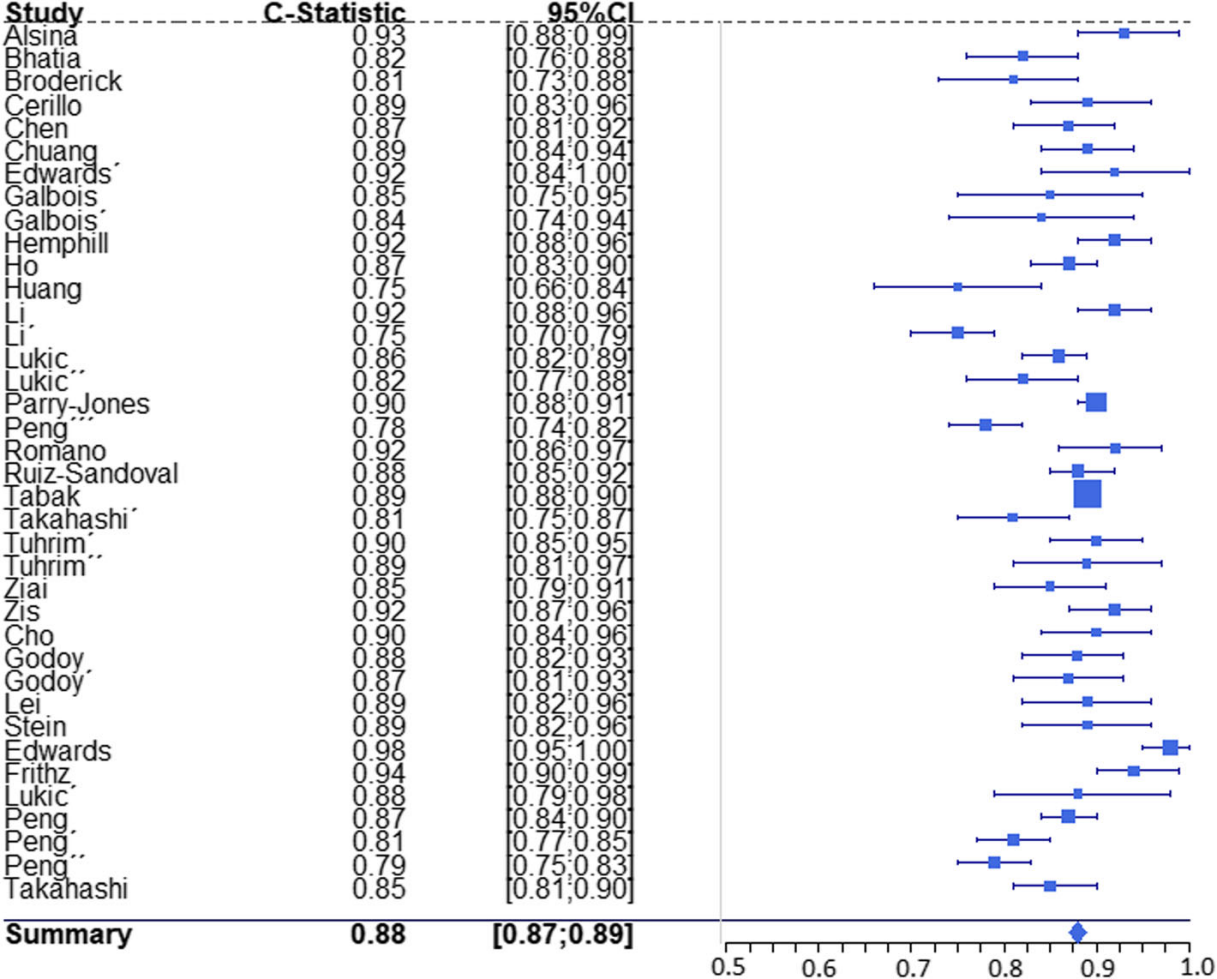
Forrest plot of reported c statistics for mortality prediction tools

**Fig. 4:**
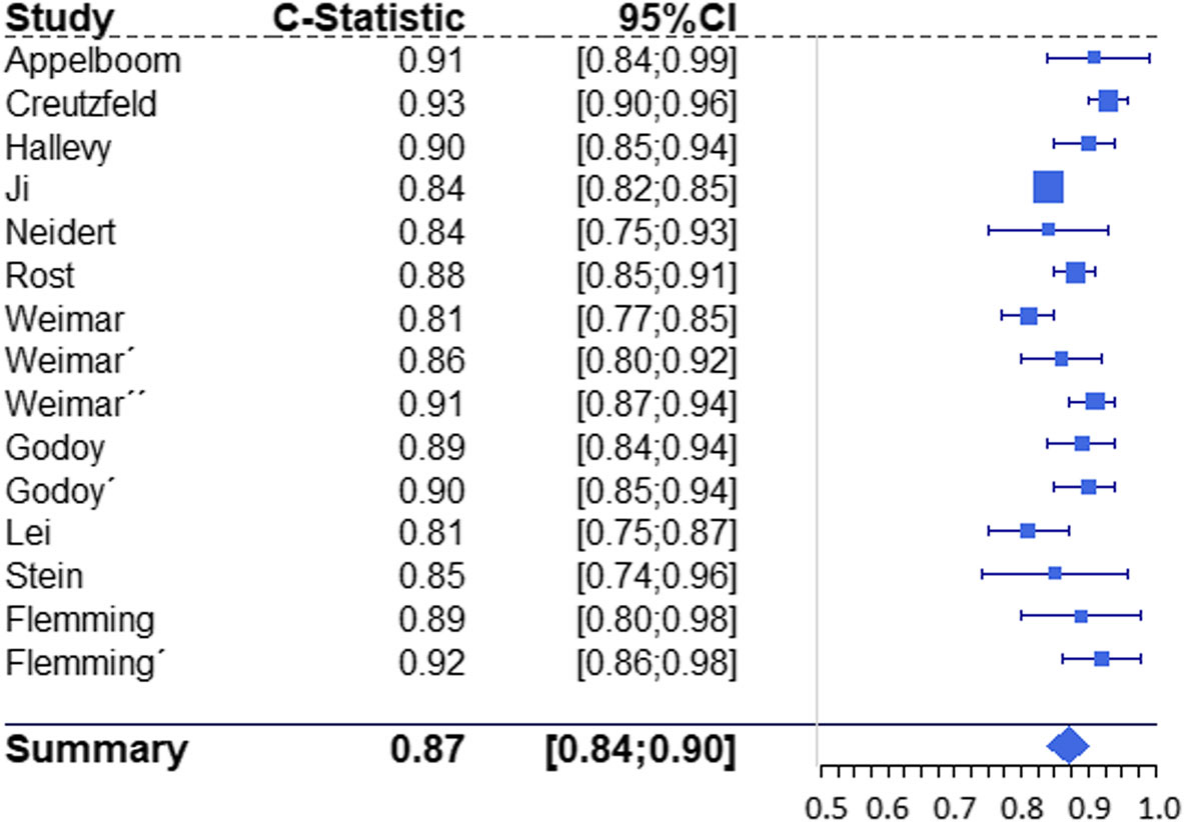
Forrest plot of reported c statistics for functional outcome prediction tools

C-statistics and respective 95% confidence intervals were retrieved from 38 mortality prediction tools and 15 functional outcome prediction tools (Table 1). Forest plots are depicted in Figs. 3 and 4. The lowest reported value was 0.745 [49] and the highest reported value was 0.984 [28]. Table 3 depicts robust variance estimates of pooled c-statistics for all tools combined and subgroup analysis for mortality prediction tools, functional outcome prediction tools, logistic regression based tools, and machine learning algorithms, along with comparisons using metaregression. All subgroups showed values for pooled c statistics > 0.80. Mortality prediction tools and machine learning algorithms showed higher pooled AUCs but the differences were not statistically significant. Other measures of discrimination reported include accuracy, reported for 22 tools [20, 24, 26–30, 33, 37, 40–42, 54–56, 63, 68], sensitivity and/or specificity, reported for 31 tools [10, 14, 16, 22, 24, 31, 34, 38, 39, 41, 43, 44, 46, 49–51, 54–56, 58, 62, 63, 68], and predictive values, reported for 22 tools [10, 14, 31, 38, 41, 43, 44, 46, 51, 52, 54, 56, 58, 62, 63]. Calibration assessment was reported using a calibration plot for three derivations [42, 59, 64], the Hosmer-Lemeshow test for 14 derivations [20, 22, 24, 28, 32, 33, 39, 47, 51, 58, 60, 63] and the Le Cessie and Howelingen test reported for one derivation [64].

**Table 3:** RVE pooled c statistics and subgroup comparisons using metaregression

Prognostic tools	Nr studies	Nr tools	Pooled c-stat	95%CI		I^2^	ß	95%CI		p
				Lower	Upper			Lower	Upper	
Overall	40	53	0·878	0·864	0·891	79%	–	–	–	–
Mortality prediction tools	30	38	0·880	0·865	0·894	80%	-0·007^a^	-0·039^a^	0·026^a^	0·679
Functional outcome prediction tools	13	15	0·872	0·842	0·901	77%				
Logistic regression based tools	37	43	0·874	0·858	0·889	76%	0·018^b^	-0·034^b^	0·070^b^	0·490
Machine learning algorithms	6	9	0·898	0·821	0·976	88%				

^a^mortality prediction tools as reference group

^b^logistic regression based tools as reference group

## Discussion

Prognostic models for ICH patients have demonstrated good discrimination in derivation studies, regardless of the outcome in question (mortality or functional outcome). These tools have been derived in different ICH populations, ranging from “general” ICH (i.e. primary or spontaneous) to more specific populations (ex. arteriovenous malformation related bleeds, dialysis patients, comatose patients). Cohort studies are the predominant study design: this design is well suited for prognostic tool derivations due to an optimal measurement of predictors and outcome [69]. Other sources of data used included registries, case-control studies, randomized clinical trial data and administrative databases. Of these, the last two raise concerns about representativeness and quality of data: on one side, clinical trials usually have the highest quality of data, but restrictive inclusion and exclusion criteria might hamper generalizability [70]; on the other side, administrative databases might allow for easy access to a large quantity of patient data, but they are prone to errors in codification, data discrepancy, and missing data [71]. A considerable number of studies (*n* = 11) were multicentric, conceding a theoretical advantage in terms of generalizability. The sampling method was frequently not reported (*n* = 15) but was consecutive for most studies, again assuring the representativeness of the population and minimizing in a convenient manner the risk of bias due to selective sampling.

Most mortality prediction tools focused on death at discharge or 1 month: this timing seems appropriate, since most deaths due to ICH occur early in the disease [1]. However, the same cannot be said for functional outcome prediction: significant changes in functional status have been described in ICH patients up to 1 year [72], rendering outcome predictions at 1 month or discharge less useful. Noticeably, 12 derivation procedures focused on functional outcome at discharge or 1 month. A reasonable compromise would be prediction at three to 6 months, allowing enough time for patient recovery without excessive loss to follow up or occurrence of competing events. Another important issue is that studies with longer follow-ups did not report on outpatient care interventions (ex. rehabilitation), making generalizability of their results less straightforward. Functional outcome prediction was mostly binary and used different scales and cut off values: whereas the optimal method of functional outcome measurement in ICH patients is debatable [73], the usage of different scales and cut offs between tool derivation studies makes comparisons between these instruments more difficult. Only five studies reported blinded outcome assessment: whereas mortality is a rather “hard outcome”, functional outcome evaluation is inherently more subjective and thus more prone to evaluation bias.

Derivation studies were rather heterogeneous in the number of patients and events analyzed. Interestingly, the four most frequently included variables for mortality prediction were also the four most frequently included variables for functional outcome prediction (Fig. 2). This overlap suggests that mortality prediction tools should, at least to some extent, predict functional outcome and vice-versa. The number of events per variable is a simple rule of thumb to assess the adequacy of sample size: it is suggested that a minimum of ten events per variable are required to prevent overfitting during statistical modelling [73], but a lower rate was found for 21 tools, although admittedly not all of them were regression based.

Missing data, whether pertaining to missing predictors or loss to follow-up, is also a potential source of bias for derivation studies, with the risk of bias relating to the amount of missing data and the extent to which it is missing at random. Handling of missing predictors was frequently not reported (22 studies). Where it was reported, complete case-analysis was the method most frequently used, which potentially creates non-random, non-representative samples of the source population. For this purpose, guidelines for prediction modelling studies have suggested preferential use of other methods such as multiple imputation, noticing however that if the number of missing predictors is extensive this technique will not be sufficient to handle this problem [69]. The same argument regarding risk of bias may be made for loss to follow-up: 4 studies reported a loss to follow-up > 10%.

Discrimination and calibration are important properties for predictive models that should be reported. Discrimination relates to the extent to which a model distinguishes those who will suffer the outcome of interest from those who will not, whereas calibration refers to the agreement between observed and predicted outcome rates [74]. C statistic is the most commonly used performance measure for discrimination [75] but it was retrieved for only 38 derivations focusing on mortality and 15 derivations focusing on functional outcome. Taken together, these studies demonstrated good discriminatory ability for both predictions. The pooled C statistic for mortality prediction was 0.880 and the pooled C statistic for functional outcome prediction was 0.872 but these results must be interpreted with caution, due to the heterogeneity in the included studies in terms of population studied, selected predictors, method of model development and choice of outcome. Other forms of discriminatory ability reported include accuracy, sensitivity/specificity, and positive/negative predictive values, but the interpretation of these measurements is less straightforward: the first two require the use of cut-off points for predicted probabilities, therefore not allowing the full use of model information, whereas the last depend on the overall probability of the event in the studied sample, hampering extrapolations for other populations with different event rates. Calibration was only reported for 14 tools, either using the Hosmer-Lemeshow test, the Le Cessie and Howelingen test, or a calibration plot.

The most frequently used method for model derivation was logistic regression. There seems to be no consensus about the best method for variable selection during multivariate logistic regression modelling, but most studies used automatic methods. These methods allow for a more efficient use of data but come with an added risk of model overfit and possible exclusion of important predictor variables due to chance, especially when sample sizes are small [76]. Nearly half of the regression based tools were simplified in the form of risk scores, allowing for an easier application. Machine learning algorithms found in our systematic review included decision trees (four), artificial neural networks (four), support vector machines (one), random forests (one) and a hybrid approach (one). These methods are an alternative to logistic regression that requires less formal statistical training and offer more efficient use of data and a higher ability to detect non-linear relations. However, they are prone to overfitting, extremely sensible to small perturbations in data and empirical in the nature of model development [77, 78]. Despite being pointed as more statistically efficient, these methods were not superior to logistic regression for discrimination in our review.

When models are tested in the same sample on which they were derived, their results tend to be biased due to overfitting: to minimize this problem, internal validation (resampling) techniques can be used. Only 19 derivations used resampling techniques for overfit adjustment. Bootstrapping is recommended as the preferred method of internal validation [74], but was performed for only two. Other methods encountered included cross-validation and split sample. The later, used in three tools, is regarded as the least effective method since it reduces statistical power for the derivation procedure and does not validate the results in a new population.

In summary, the results from our review suggest that the most promising prognostic tools are i) logistic regression based risk scores, which combine the high discrimination showed by logistic regression with the ease of application typical of prognostic scores; ii) derived from general cohorts (i.e, spontaneous or primary ICH) to maximize generalizability; iii) without significant loss to follow up, to minimize risk of bias; iv) with early outcome measurement for mortality (i.e, discharge or 1 month) and later outcome measurement for functional outcome (i.e, 3 months or more) and v) showing high discrimination with an appropriate EPV rate. Examples of such scores include the scores by Chen [45], Hemphill [48], Ho [64], Romano [56] and Ruiz-Sandoval [58] for mortality, Ji [51] and Rost [57] for functional outcome prediction and Godoy [62] for a combined outcome. Not surprisingly, several validation studies have been published for these tools. Other factors to take into account are internal validation and blinded outcome assessment, the latter being particularly important for functional status.

Our review has limitations. Firstly, there were no clear guidelines on conducting and reporting studies for prognostic tool derivation at the time most of these studies were performed. This lead to frequent underreporting and higher difficulty in retrieving information about important methodological aspects and performance measures, which reflected on the results of our review. As an example, we were only able to retrieve c-statistics for 53 derivations, which means that several tools could not be evaluated for this important discrimination measure. Guidelines have recently been published to give guidance on this issue [69]. Second, studies have demonstrated that healthcare professionals are frequently pessimistic in the face of neurological emergencies [79]. This negative perception can result in a “self-fulfilling prophecy”, whereby the physician’s perception will lead to early withdrawal of care which, by itself, will facilitate a negative outcome [79]. Most studies assessing the effect of early care limitation in the performance of prognostic models have focused on validation studies [47, 80, 81]. According to these studies, models underestimate adverse outcomes in patients with early care limitation and overestimate in patients without. However, care limitation has also been demonstrated to be an independent predictor of poor outcome [34, 82]. Hence, one should expect that withdrawal of care would affect model performance also in derivation studies, but this factor was not taken in to account in the majority of studies included in this review. A possible solution for this problem is to derive prognostic models from patient populations with maximum level of care. Such approach was more recently used by Sembill and collaborators to derive the max-ICH score [83]. Third, the previously discussed aspects of prognostic tool derivation are useful to assess the risk of bias and external validity of these instruments, but they do not necessarily determine the way these tools will behave in clinical practice. Risk of bias does not necessarily imply existing bias, and the ultimate issue is how they behave in an independent external dataset [84]. At the time of our search we identified external validation studies for only 27 prognostic tools [14, 16, 20, 22, 26, 27, 29–31, 37, 40, 41, 43–46, 48, 54, 56–59, 62, 63]. Nevertheless, derivation studies less prone to bias are more likely to perform well in validation studies. The issues discussed in this systematic review should then be taken as a guidance for future studies seeking to validate existing prognostic tools or to derive new ones in ICH patients as well as in other populations.

## Conclusions

Prognostic models showed high discrimination in derivation studies for mortality and functional outcome prediction in ICH patients but numerous methodological and reporting deficiencies were present, namely insufficient length of follow-up for functional outcome, absence of blinding, reporting and handling of missing data, low EPV rate, infrequent use of appropriate internal validation procedures and underreporting of important model performance measures. Machine learning methods have not proven to be superior to regression based models and a significant number of these tools weren’t submitted to external validation. Guidelines have been reported to support authors in developing and reporting studies both for prognostic model derivation and validation [69].

## Additional file

Additional file 1: Search syntax used for identification of candidate studies. (DOCX 1028 kb)

## Abbreviations

AUC: Area under the ROC curve
CI: Confidence interval
EPV: Event per variable
ICH: Intracerebral hemorrhage
ROC: Receiver operating characteristic
RVE: Robust variance estimation
