# Supplementary material for: Prognostic models for intracerebral hemorrhage: systematic review and meta-analysis
Source: BMC Med Res Methodol. 2018 Nov 20;18:145. doi: 10.1186/s12874-018-0613-8 (PMC6247734; doi:10.1186/s12874-018-0613-8)
Supplement: Supplementary file 1 — Search syntax used for identification of candidate studies. (DOCX 1028 kb) [file 12874_2018_613_MOESM1_ESM.docx]

**Additional file: Search syntax used for identification of candidate studies.**
